# First experience with augmented reality neuronavigation in endoscopic assisted midline skull base pathologies in children

**DOI:** 10.1007/s00381-021-05049-3

**Published:** 2021-01-30

**Authors:** Valentina Pennacchietti, Katharina Stoelzel, Anna Tietze, Erwin Lankes, Andreas Schaumann, Florian Cornelius Uecker, Ulrich Wilhelm Thomale

**Affiliations:** 1 Pediatric Neurosurgery, Charité-Universitätsmedizin Berlin, Campus Virchow Klinikum, Augustenburger Platz 1, 13353 Berlin, Germany; 2 Department of Otorhinolaryngology, Charité-Universitätsmedizin Berlin, Berlin, Germany; 3 Institute of Neuroradiology, Charité-Universitätsmedizin Berlin, Berlin, Germany; 4 Department for Pediatric Endocrinology and Diabetes, Charité-Universitätsmedizin Berlin, Berlin, Germany

**Keywords:** Endoscopic assisted endonasal approach, Neuronavigation, Sellar region, Neuroendoscopy

## Abstract

**Introduction:**

Endoscopic skull base approaches are broadly used in modern neurosurgery. The support of neuronavigation can help to effectively target the lesion avoiding complications. In children, endoscopic-assisted skull base surgery in combination with navigation systems becomes even more important because of the morphological variability and rare diseases affecting the sellar and parasellar regions. This paper aims to analyze our first experience on augmented reality navigation in endoscopic skull base surgery in a pediatric case series.

**Patients and methods:**

A retrospective review identified seventeen endoscopic-assisted endonasal or transoral procedures performed in an interdisciplinary setting in a period between October 2011 and May 2020. In all the cases, the surgical target was a lesion in the sellar or parasellar region. Clinical conditions, MRI appearance, intraoperative conditions, postoperative MRI, possible complications, and outcomes were analyzed.

**Results:**

The mean age of our patients was 14.5 ± 2.4 years. The diagnosis varied, but craniopharyngiomas (31.2%) were mostly represented. AR navigation was experienced to be very helpful for effectively targeting the lesion and defining the intraoperative extension of the pathology. In 65% of the oncologic cases, a radical removal was proven in postoperative MRI. The mean follow-up was 89 ± 79 months. There were no deaths in our series. No long-term complications were registered; two cerebrospinal fluid (CSF) fistulas and a secondary abscess required further surgery.

**Conclusion:**

The implementation of augmented reality to endoscopic-assisted neuronavigated procedures within the skull base was feasible and did provide relevant information directly in the endoscopic field of view and was experienced to be useful in the pediatric cases, where anatomical variability and rarity of the pathologies make surgery more challenging.

## Introduction

The endoscopic-assisted endonasal approach to the skull base is rarely applied in children compared to adults. In recent years, a number of authors have described the use of a transcribriform/transtuberculum/transsphenoidal or transclival access to midline skull base pathologies. In fact, they are described as anatomically, clinically, and technically challenging. Nowadays these extracranial approaches are preferred in the adult population mainly due to its minimal invasive character, with a few exceptions and contraindications such as anatomical anomalies, for example, “kissing carotids”, or particular configuration of the lesions. The same cannot always be applied to children, although in recent years, the technique has been used increasingly in some pediatric cohorts [22, 24, 25]. Specifically, the incomplete pneumatization of the sphenoid sinus, the thickness of the bone and the intercarotid distance in pediatric skull base anatomy influence the surgical planning to calculate the distance to the structures such as planum sphenoidale, tuberculum sellae, dorsum sellae, or clivus [26]. In addition, the technique in ventral approaches to the skull base, which is usually linked to lower morbidity, is more complex in children, consequently with higher incidence of cerebrospinal fluid (CSF) leak [16]. The access through the piriform aperture might have also implications on the growth of the midface in case of excessive bone damage in younger children [1, 14].

The implementation of neuronavigation into endoscopic procedures can help to better identify anatomical variations, the lesion’s boundaries within the surgical field, and spatial relations between lesion and normal tissue. In conventional neuronavigation systems, the information must be transformed from the navigation workstation by the surgeon, while the endoscope enables pointer function after registration. New perspectives can be reached by implementing augmented reality (AR) technologies in neuronavigation systems. This has already been used in microscopic-navigated neurosurgery, in which navigated information can directly be superimposed into the surgical field of view [2, 6, 26]. The integration of AR into navigated endoscopy is specifically meaningful since the endoscopic view is somehow limited but can be brought directly into the surgical field. The connection between real imaging and virtual data, presented as contour reconstructed objects onto real anatomical views, has been described to be helpful especially in minimal invasive surgical techniques [17]. The technology enhances the definition of targets and their spatial relationships to neighboring structures. Such advancements are particularly relevant in the so called tunnel principle of endoscopic surgery, although operator-related and system-borne biases still limit their routine use [3].

The objective of this paper is to retrospectively report our experience, with regard to morbidity in the field of endoscopic skull base surgery performed in combination with AR in the pediatric population.

## Patients and methods

We conducted a retrospective analysis on 17 endoscopic procedures in which AR-enhanced neuronavigation was used. The interventions took place in our center in a total of 11 patients, using the transnasal corridor in all but one case, in which the transoral corridor was utilized, to approach lesions of different nature affecting the midline skull base. The cohort of patients was treated in a period between October 2011 and May 2020 in an interdisciplinary setting. The clinical files of every patient were evaluated, given all the characteristics of the cohort as described in Table 1.

**Table 1:** Patient characteristics (PD, progressive disease; CR, complete remission; SD, stable disease)

Patient	Procedure	Sex	Age at surgery(years)	Diagnosis	Approach	Symptoms	Previous Surgery/Radiation	Surgery time (minutes)	Hospital stay (days)	Extent of resection (Gnekow et al, 2019)	Complications	Outcome	Follow-up (months)
**I.**	**1.**	M	13,03	Adamantinomatous Craniopharyngioma	Endonasal	Progression, visual impairment, panhypopituitarism	3/2	195	9	2c	–	PD	253,33
	**2.**	M	15,45	Adamantinomatous Craniopharyngioma	Endonasal	Progression, visual impairment, panhypopituitarism	7/3	161	7	3	Abscess	–	–
	**3.**	M	15,83	Abscess	Endonasal	Meningeal signs	8/3	27	12	2c	–	–	–
	**4.**	M	19,82	Adamantinomatous Craniopharyngioma	Endonasal	Progression, visual impairment, panhypopituitarism	10/3	140	7	2c	–	–	–
**II.**	**5.**	M	12,29	Adamantinomatous Craniopharyngioma	Endonasal	Progression	6/1	225	4	2c	–	CR	211,83
**III**	**6.**	M	11,87	Fibrous benign tumor	Endonasal	Right V neuralgia	–	106	6	Biopsy	–	SD	8,17
**IV**	**7.**	M	16,38	Aneurysmatic bone cyst	Transoral	X-XII palsy	2/0	138	9	3	–	SD	48,17
**V**	**8.**	M	10,85	Germinoma	Endonasal	Diabetes insipidus	–	113	7	Biopsy	–	CR	116,08
**VI**	**9.**	M	15,78	Rathke cleft cyst	Endonasal	Headache	–	193	7	2b	–	CR	18,17
**VII**	**10.**	F	11,42	Osteochondromyxoma	Endonasal	Epistaxis	–	258	9	1	CSF leak	CR	86,58
	**11.**	F	12,96	Iatrogenous CSF leak	Endonasal	Rhinoliquorrhea	1/0	148	6	–	–	CR	–
	**12**	F	16,60	Myxoma	Endonasal	Progression	2/0	115	7	Biopsy	–	SD	–
**VIII**	**13.**	F	12,42	Rathke cleft cyst	Endonasal	Headache	–	110	24	1	CSF leak	CR	71,92
**IX**	**14.**	F	15,37	Papillary Craniopharyngioma	Endonasal	Headache	–	166	15	2b	–	CR	58,08
**X**	**15.**	F	17,37	GH-secreting adenoma (Knosp grade III left)	Endonasal	Amenorrhea, visual impairment	–	122	7	Biopsy	–	x	x
**XI.**	**16.**	F	14,45	Rathke cleft cyst	Endonasal	Headache	–	151	6	2c	–	SD	8,17
	**17.**	F	14,98	Rathke cleft cyst	Recurrence with hemorrhage in follow-up MRI		1/0	126	5	1	–	CR	12

### Preoperative procedure

**Fig. 1:**
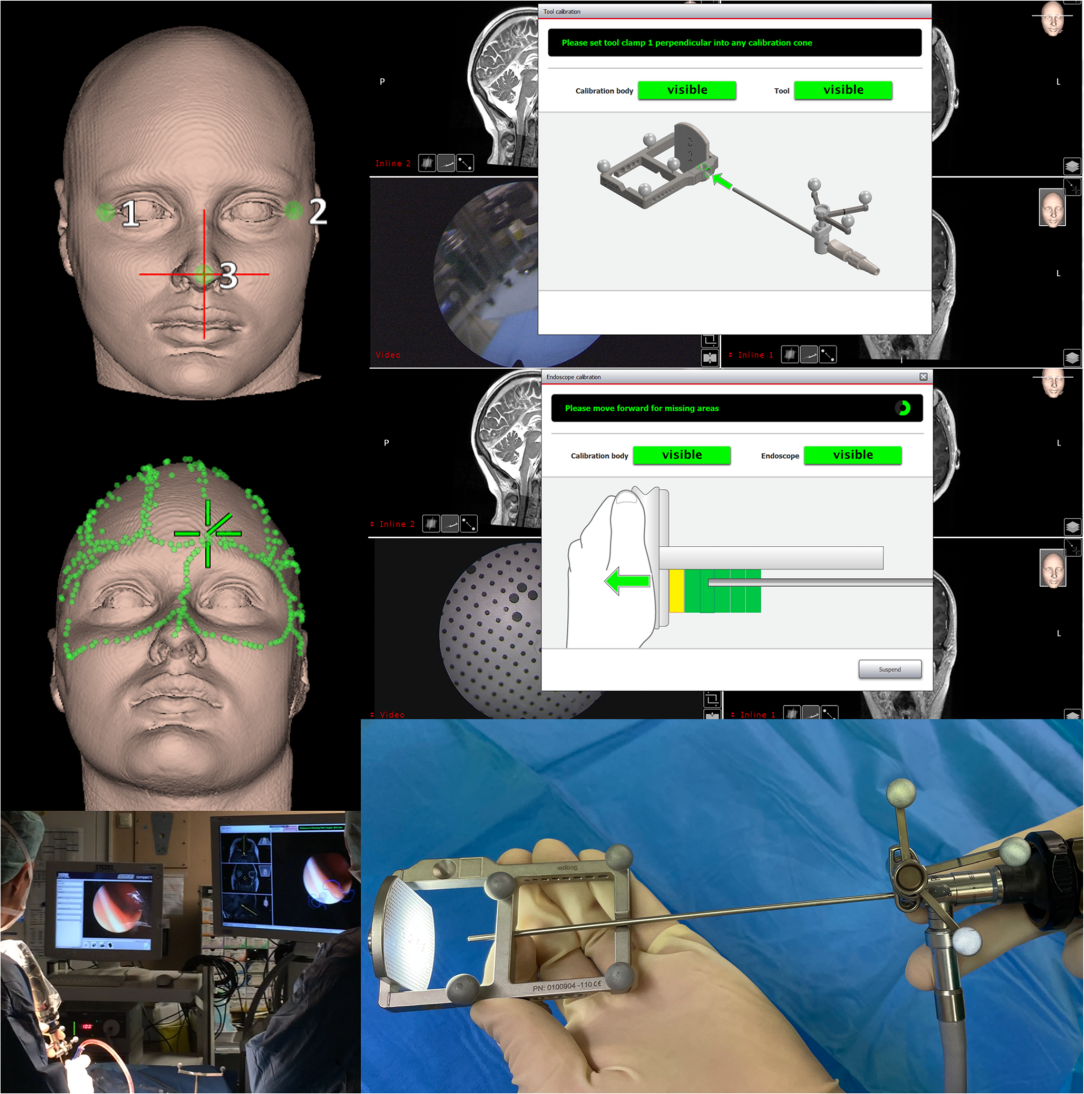
Hybrid registration procedure of the patients head before surgery capturing anatomical landmarks and multiple random points on the surface of the nose, the forehead and the anterior calvarium (left upper and middle pictures). The registration of the endoscope includes two steps using a registration matrix in which the length of the endoscope was defined and the visual field was registered at a specific magnification and camera orientation using a predefined matrix pattern (right upper, middle and lower pictures). In the intraoperative setting both, the endoscope’s and neuronavigation screen could be appreciated in parallel. The endoscopic views on the navigation screen incorporates the AR information together with the reconstructed 3D MRI sections along the endoscopes orientation (left lower picture)

**Fig. 2:**
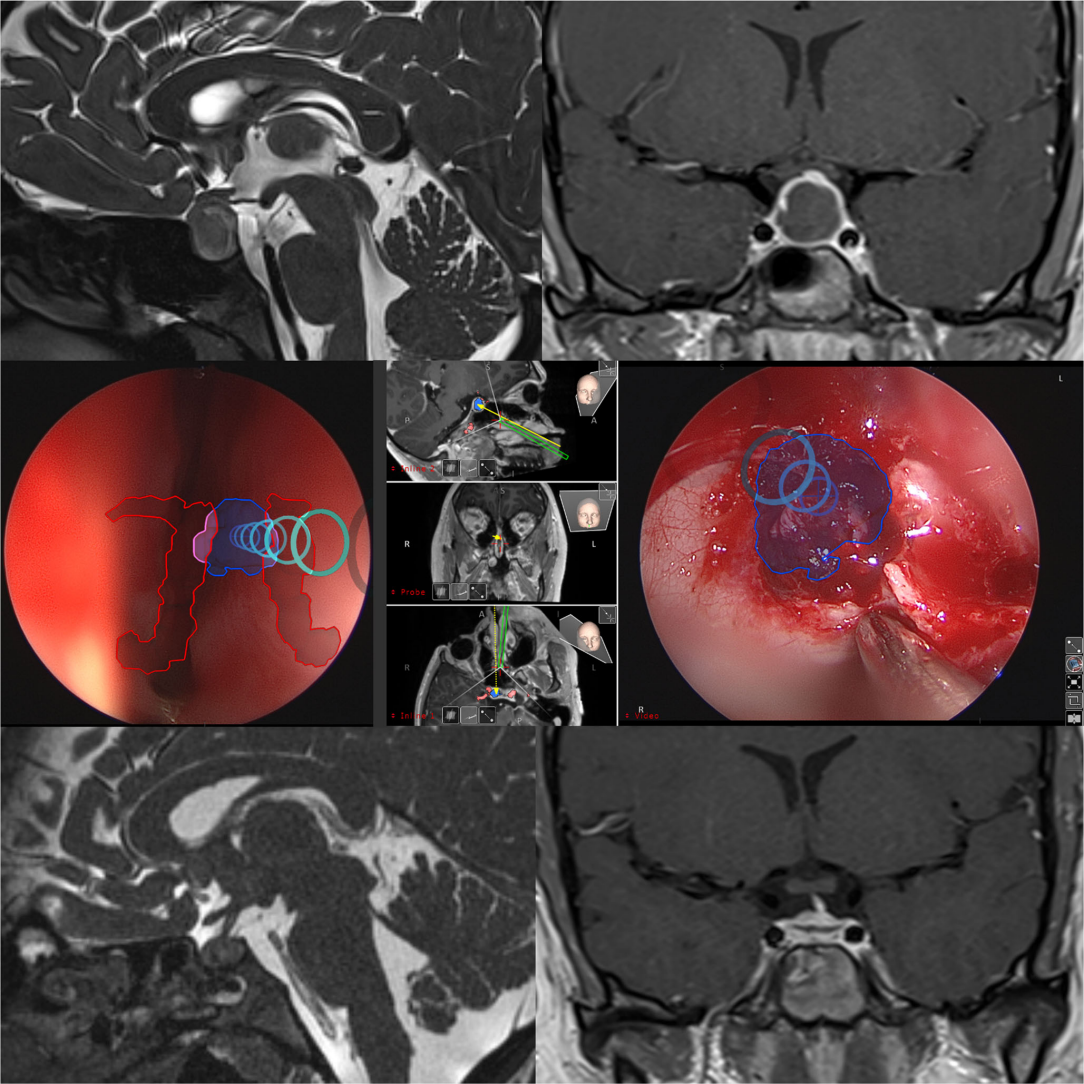
Case example of a patient with a Rathke cleft cyst: upper row: preoperative MRI (T2 sagittal and T1 post-gadolinium coronal sequence). Middle row: intraoperative endoscopic view with AR information indicating the trajectory (multiple rings), the target (blue contour) and the carotid arteries. Lower row: postoperative MRI (T2 sagittal and T1 post-gadolinium coronal sequences)

**Fig. 3:**
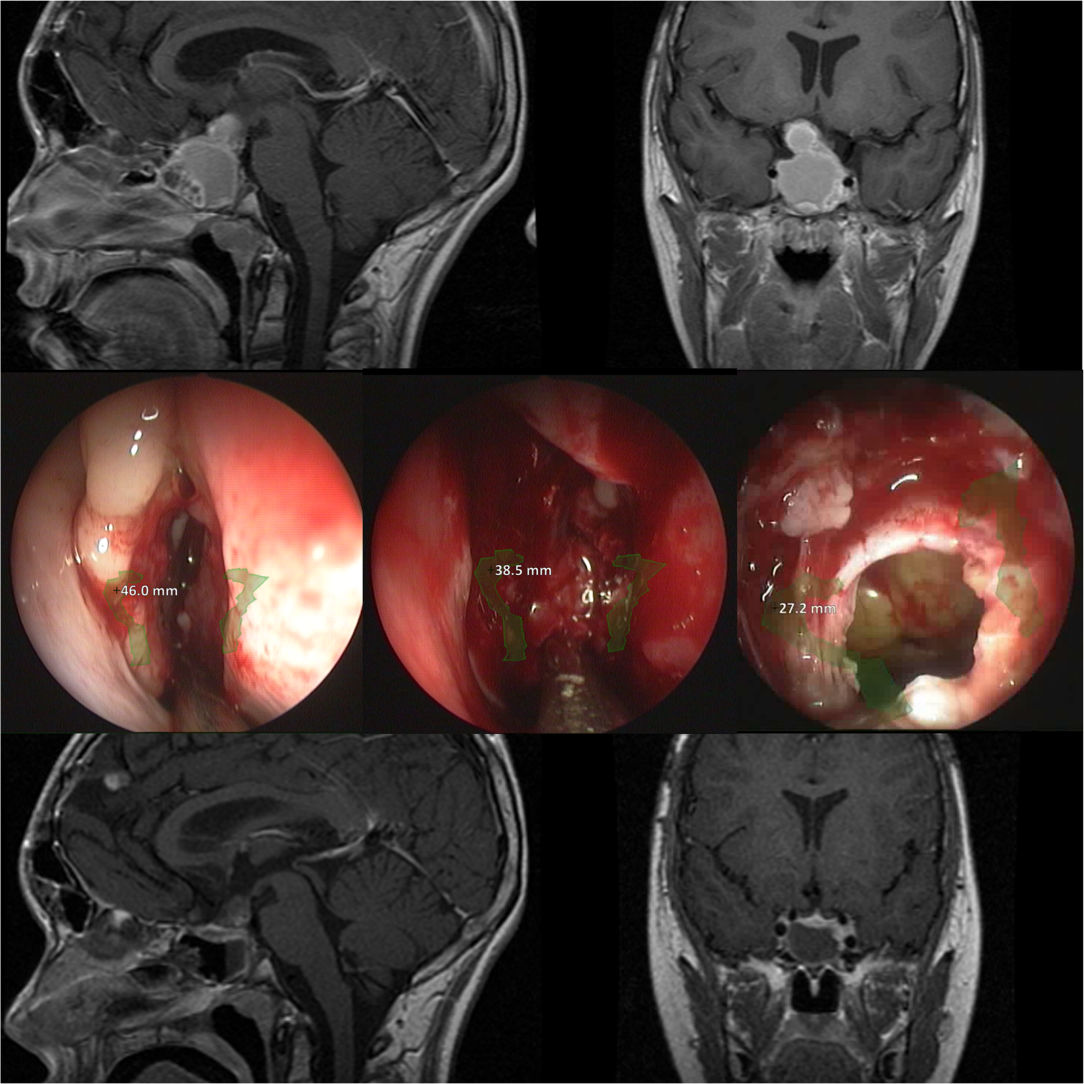
Case example of a patient with a craniopharyngioma: Upper row: preoperative MRI (T1 sagittal and coronal post-gadolinium sequences). Middle row: intraoperative endoscopic view at different anatomic levels of the turbinates, the sphenoid cavity and the sella with AR information indicating the carotid arteries. Lower row: postoperative MRI (T1 sagittal and coronal post-gadolinium sequences)

The surgical treatment using navigated endoscopic-assisted surgery was indicated in cases with localized skull base lesions without relevant extension to the surrounding structures. All the patients underwent preoperative MRI with volumetric high-resolution 3 Tesla scans (Skyra 3 T Siemens system, Siemens, Erlangen, Germany), usually importing 3D T1-weighted (MPRAGE, isotropic resolution of 1 mm) and 2D T2-weighted (2–3 mm slice thickness) sequences in the planning workstation NovaPlan (version 2.6.10, Scopis, Germany). Bony structures could mostly be identified from those fused MRI sequences. CT scans and thus radiation exposure could therefore be avoided in most of the patients. The lesion as well as target and anatomical structures at risk were outlined within the planning tool in each section for volume segmentation. In addition, the optimal trajectory towards the target was defined. The surgical plan was transferred to the navigation system, and the patient’s head was fixed in a Doro pediatric head fixation system (PMI Surgical, Freiburg, Germany). The spatial orientation of the head was identified by a hybrid anatomical landmark and surface matching registration procedure. The endoscope was integrated into the navigation by using an optical reference frame to define the geometric dimension (length and diameter). In addition, the visual field of the endoscope was registered by identifying a predefined pattern of a reference matrix from variable distances (Fig. 1). For the latter step, it is important that the magnification and orientation of the camera on the endoscope remains stable during AR navigation. The endoscope’s video that was transferred to the neuronavigation system and the trajectories, the target lesions, and regions at risk were presented in the endoscope’s view (Figs. 2 and 3).

### Surgical technique

Every surgery was performed in collaboration with an ear-nose-throat (ENT) specialized surgeon in an interdisciplinary team. For the approach, the head of the patient was positioned with slight extension of the neck and minimal contralateral deviation to the entry side. An endoscope (Hopkins optic, Karl Storz, Tuttlingen, Germany) with a zero degree vision was used. After disinfection, sterile covering of the surgical field, and naphazoline preparation of the nasal mucosa bilaterally, the ENT surgeon gently deviated the inferior and middle turbinates on one nostril until the identification of the sphenoidal ostium, at the level of the superior turbinate and cranial to the nasal choana. The mucosa was dissected only at the very end, and the ostium was opened in order to remove the anterior wall of the sphenoid sinus and the septal sphenoid structures. At this point, when the window was large enough, the neurosurgeon identified, under the guidance of the neuronavigation system, the bony boundaries and landmarks of the sellar region at the posterior cranial wall of the sphenoidal sinus, such as the carotid arteries, optic nerves, tuberculum sellae, optico-carotid recesses, and dorsum sellae. By drilling the bone towards the target, the dura of the sellar region was exposed coagulated and incised. The lesion was resected under endoscopic view in a bimanual technique according to its extension, as defined by the underlying pathology. All procedures but one applied the endonasal approach. Only in one case, an aneurysmatic bone cyst located in the upper third of the clivus, the approach was transoral. The cyst was additionally embolized preoperatively using endovascular techniques. In this case, only the soft palate was dissected to appropriately reach the lesion.

At the end of the procedure, a closure with gelatin sponge covered with a fibrinogen and thrombin matrix (TachoSil, Takeda Austria, Austria) as well as additional fibrin glue was performed. In the cases of CSF leakage, a combination of a dural substitute, naso-septal flap, and TachoSil was used. Finally, a nasal tamponade was applied bilaterally for 48–72 h.

#### Postoperative evaluation

The extent of resection and potential perioperative complications were documented by an early MRI 24–48 h after surgery. The intensive care unit (ICU) and hospital stay, as well as the postoperative long-term outcome, were evaluated by reviewing the clinical charts from the hospital stay and outpatient visits, accordingly.

## Results

In a total of 11 patients (5 females), 17 endoscopic AR-navigated endoscopic procedures were conducted and retrospectively analyzed. Their mean age at surgery was 14.5 ± 2.4 years (range: 10.8–19.8 years). Histopathological diagnoses included five craniopharyngiomas (four adamantinomatous, one papillary), three Rathke cleft cysts, one GH-secreting macroadenoma, one myxoma, one germinoma, one aneurysmatic bone cyst, one abscess, one CSF leak, one benign fibrous lesion, and one osteochondromyxoma. Reoperations with AR navigated endoscopic surgery were performed in one craniopharyngioma (4 surgeries), in the myxoma patient (3 surgeries), and in one recurrent Rathke cleft cyst. Mean time to recurrent surgery was 2.1 ± 1.5 years. Five patients received previous surgeries before AR-navigated endoscopic-assisted intervention (craniopharyngiomas, *n* = 2; aneurysmatic bone cyst with pharyngeal extension; Rathke cyst; myxoma; *n* = 1 each; Table 1).

Signs and symptoms that indicated intervention were disease progression during regular follow-up imaging in 5 cases and lesion-related symptoms in 5 cases (headaches, dizziness, trigeminal neuralgia, diabetes insipidus, swallowing disorders, and hypoglossal palsy). In one case rhinoliquorrhea and meningitis at 6 months after previous surgery led to the diagnosis. In another case, the lesion was identified during regular follow-up of a Carney syndrome complex (CNC).

The mean duration of the surgery was 146.7 ± 52.6 min (range: 27–258 min). All the procedures were performed integrating the use of the endoscope with augmented reality-assisted neuronavigation. This functionality was experienced in all cases as accurate. Lesions were targeted as planned in a very straightforward manner since the information was directly displayed on the surgeon’s endoscopic view within the neuronavigation workstation. Targeting was continuously updated by automatically reconstructing MRI sections along with the directions of endoscopic view. This functionality was experienced as extremely helpful in all surgeries for better orientation during the approach as well as estimation of the extension of the targeted lesion.

Postoperatively, the clinical observation and monitoring were established in the pediatric intensive care unit (ICU) usually for 24 h. In one case, the patient stayed 48 h in the ICU in order to manage possible endocrinologic alterations (e.g., hypopituitarism). In two cases, an ICU monitoring was not judged to be necessary. The mean total hospital stay duration was 8.6 ± 4.7 days (range 4–24 days).

In 9 out of 14 oncological lesions in this series, the early postoperative MRI has proven a complete or near complete removal of the lesion (extent of resection type 1: (*n* = 2), type 2b: n = 2), type 2c: *n* = 5, [13]). Partial resection was achieved in two interventions, both of which had also been intended for partial resection only. Biopsy was planned and successfully performed in four surgeries.

The observed complications comprised one postsurgical abscess in a craniopharyngioma case at 20 weeks after surgery, which was successfully treated by AR-navigated endoscopic-assisted surgery and antibiotic therapy, accordingly. Two CSF leaks required a revision surgery at 1 week and 6 months after initial surgery, respectively. One patient was surgically revised subsequently and did not show any further long-term issues. The second patient, diagnosed with a Carney Complex Syndrome, turned out to suffer from additional idiopathic intracranial hypertension with headaches and papilledema and received a ventriculo-peritoneal shunt during further follow-up.

The neurological outcome comprised one case of swallowing disorders in the patient with the aneurysmatic bone cyst, who postoperatively improved. It became possible to remove the tracheal cannula and who was slowly able to gain weight through oral nutrition. One case of residual quadrant anopia and an optic atrophy with left abducens palsy in craniopharyngioma cases remained stable after surgery and were related to the primary pathology. In the case with the GH-macroadenoma, papilledema and bitemporal hemianopsia resolved after decompressive surgery. One patient complained about regressive, but periodical headache during long-term follow-up. None of the cases did show any postoperative neurological deficit.

The endocrinological status of the patients was severely compromised before surgery, with panhypopituitarism in three patients (2 craniopharyngiomas and one germinoma). All these patients required hormone substitution already preoperatively. One patient in the series presented with the signs of growth hormone excess, high IGF-1 values (765 ng/ml), and amenorrhea before surgery (the GH-secreting adenoma). In one patient with a Rathke cleft cyst, a moderate hyperprolactinemia (98.2 mcg/l) was observed. The remaining patients did not show any signs or symptoms of endocrine dysregulation preoperatively. After surgery, panhypopituitarism was observed in the same three patients in whom it was present preoperatively, the hyperprolactinemia was normalized in the Rathke cyst patient, and the growth hormone excess was relieved (also the amenorrhea). One craniopharyngioma patient showed symptoms of diabetes insipidus with polyuria and hypernatremia, which was treated with administration of desmopressin necessary only for a short period of time. In summary, there was no significant change in the endocrine situation in comparison to the preoperative status.

The mean follow-up was 89.1 ± 79.4 months (range: 12–253.3 months). One patient needed further transcranial surgery during follow-up for other tumor locations. In two cases, a post-surgical radiotherapy was indicated, which was decided by the multidisciplinary pediatric neurooncological team, because of persistent tumor and ongoing disease dynamics (one craniopharyngioma and one germinoma). The patient with the germinoma required additional chemotherapy according to protocol guidelines. The aneurysmatic bone cyst was subsequently treated with a targeted therapy. For the entire cohort, we registered one ongoing progressive disease (craniopharyngioma), 4 stable diseases, and 8 complete remissions.

## Discussion

Endoscopic-assisted endonasal surgery has been increasingly employed over the past two decades, and its development was mainly focused on providing better optics and improved instruments and accelerating the learning curve of young surgeons. In terms of visualization technologies, high-resolution image acquisition and 3D technology applied to the endoscopic vision have been developed [27, 29]. Furthermore, in order to enhance the orientation during surgery the combination of navigation together with endoscopy is established; however, being limited to pointer navigation functionality during surgery. Our study reports for the first time experience of using augmented reality (AR)-assisted navigated transnasal endoscopic neurosurgical procedures in a pediatric patient cohort. With the current study, we have shown feasibility of the technology and improvement in better presentation of relevant surgical information to surgeon during intervention. Using the same technologies in the context of intraventricular endoscopic interventions, we were previously able to proof accuracy of the procedure [12]. The use of AR in skull base surgery has previously been described in microsurgical procedures [2, 8, 28]. A recent meta-analysis on the subject has shown that the limited amount of experience so far does not allow conclusions to be drawn about the superiority of conventional neuronavigation compared to AR neuronavigation systems [11]. The latest applications of AR in cranial surgery even include calvarial remodeling in children with craniosynostosis [15] and vascular neurosurgery [23]. To date, the use of AR in endoscopic transnasal approaches has been studied only in ENT surgeries [3, 30, 31]. As already described by Winne et al. [30] from the ENT’s point of view, overlay visualization of the elaborated surgical target and the navigation-calibrated endoscope represent a valuable and safe instrument to improve the surgical performances in endoscopic endonasal procedures. The intraoperative support consists of the simultaneous visualization of the endoscope’s position on standard axial, sagittal, and coronal MRI views as well as trajectory-aligned reconstruction of MR imaging on the navigation screen. At the same time, the endoscope’s screen displays the anatomical landmarks, thus offering the possibility to check the precision of the plan. AR combines the information of the two sources, the video and the navigation making it possible to directly relate the surgical field and to the preoperative planning overcoming the additional effort of interpreting MRI data in relation to the surgical field [3, 9, 12, 17].

Until now there are only limited reports in the literature about the combination of navigation and AR to enhance transnasal endoscopic neurosurgery. The largest cohort consisting of 313 adult patients was described by Caversaccio et al. [3] and was mainly composed of nasal pathologies. A more recent study covers 134 anterior skull base endoscopic procedures (from pituitary adenomas to sinusal pathologies) in an adult population [31]. Other authors focus their preliminary work on cadaveric studies of 14 and 15 specimens, respectively, in which endoscopic endonasal approaches were combined to the use of an AR navigation tool [9, 20]. Two further studies report experiences on 12 and 5 adult patients, treating pituitary and craniocervical junction pathologies, respectively [7, 17] (Table 2).

**Table 2:** Augmented reality neuronavigation and endoscopic skull base surgery

Study	Year	N. of cases	Group	Pathology
Kawamata T et al. [17]	2002	12	Adults	Pituitary tumors (9 adenomas, 1 craniopharyngioma, 1 Rathke’s cleft cyst, 1 chordoma)
Caversaccio M et al. [3]	2007	313	Children, Adults	Mainly naso-sinusal pathologies (181 polyposis and sinusitis, 29 biopsies, 29 frontal sinus surgeries, 22 tumors, 18 sphenoidal sinus surgeries, 11 mucocele, 8 choanal atresia, 7 CSF leak, 6 cystic fibrosis, 1 embolization, 1 crista galli cyst)
Dixon BJ et al	2013	14	Cadavers	–
Choudhri O et al [7]	2014	5	Adults	Craniocervical junction pathologies
Li L et al [20]	2016	15	Cadavers	–
Zeiger J et al [31]	2020	134	Adults	Pituitary tumors (68); other tumors, CSF leaks, sinonasal pathology (66)

Literature reports on children treated by means of an endoscopic skull base approach are sparse, with the largest study consisting of 133 patients [5], followed by a 28-patient retrospective study [8], both of which describing a relatively homogeneous spectrum of pathologies. As already discussed in relation to complications, in fact, the most common endoscopically treated conditions that are described are encephalocele, meningoencephalocele [4, 8, 10, 22, 24], and pituitary adenomas [5, 16, 21, 25]. It is, however, described that a broader range of pathological entities results in a higher rate of complications [5, 18]. Whether the endoscopic method is preferred over the microscopic technique depends mainly on the surgeon’s experience and comfort. Both choices, when routinely performed, are associated with comparable rates of success and complications [19].

Generally, the rate of complications in endoscopically treated children with sellar lesions appears to be higher compared with adults. This may be due to the pathology spectrum involving the sellar and parasellar regions in children is quite variable and our cohort reflects this heterogeneity. The histopathological diagnosis comprises rare and sometimes relatively aggressive diseases. We observed that two out of three patients in our series with postoperative issues had a long history of multiple surgeries and repeated recurrence of the underlying pathology. In one case, a patient with a craniopharyngioma with multiple previous transcranial surgeries as well as radiotherapy sessions developed a local abscess, which was treated successfully by evacuation and antibiotic therapy. The other patient was operated for an osteochondromyxoma and developed a CSF leak after surgery that was handled with an endoscopic endonasal repair through a turbinate mucoperiosteal flap. The patient turned out to have an idiopathic intracranial hypertension requiring a ventriculo-peritoneal shunt during follow-up. In our series, long- and short-term neurological complications as well as nasal disturbances were not observed, similarly to other experiences reported in literature. A multidisciplinary approach together with ENT surgeons focuses on the goal to keep the approach as minimal invasive as possible.

## Conclusion

The use of AR-assisted neuronavigation in endoscopic skull base approaches is representing a helpful adjunct, which has not yet been routinely and systematically introduced in this neurosurgical field. This application appears to be particularly beneficial when performing endoscopic surgery in children, because of the variable vascular, nervous and bony anatomy, as well as rare pathologies in this population. Further investigation, also articulated with more homogenous pathologies, is necessary to prove its benefits for the clinical outcome of the patients. The technique is not widely available yet and needs further development to enable routine use on a broader basis.
